# Medical News and Items

**Published:** 1877-09

**Authors:** 


### Medical News and Items.

Dr. Lewis A. Sayre, of New York, is entertaining the medical public of Great Britain with his description of the plaster of Paris treatment of spinal curvature.

---

The Chicago Society of Physicians and Surgeons has formally accepted the invitation of the Librarian of the Medical Press Association, and will hereafter meet in the rooms of the latter organization, at No. 188 S. Clark st.

LICENTIATES OF THE STATE BOARD OF HEALTH.—We propose to publish, for the benefit of our readers, the names and dates of graduation, of the physicians in the State and City, licensed to practice medicine by the State Board of Health.

---

PROF. ALPHEUS BENNING CROSBY, M. D., died August 10th of apoplexy, at his country-seat, Hanover, N. H. He held the Professorship of Anatomy at Bellevue Hospital Medical College and of Surgery at Dartmouth. He had also lectured on surgery at Michigan University and Long Island Medical College. He was unusually gifted as a lecturer, and enjoyed a popularity not more because of his rare ability, than of his genial social qualities. All who knew him will be pained to learn of his untimely death.

---

INDIANA ILLINOIS AND KENTUCKY INTER-STATE MEDICAL SOCIETY.—This Society will hold its third session in the city of Evansville, Ind., commencing 11 A. M., Third Tuesday in October, 1877, and continue three days.

President, W. H. Byford, A. M. M. D., Chicago, Ill.
1st Vice President, C. L. Dismukes, M. D., Mayfield, Ky.
2d Vice President, G. G. Barton, M. D., Washington, Ind.
3d Vice President, F. W. Deming, M. D., Pana, Ill.
Recording Secretary, G. W. Barton, M. D., Mitchell, Ind.
Corresponding Sec'y., F. W. Beard, M. D., Vincennes, Ind.
Treasurer, A. Patton, M. D., Vincennes, Ind.

Section of Surgery and Anatomy, J. W. Thompson, M. D., Paducah, Ky., Chairman; J. H. Letcher, M.D., Henderson, Ky., Sec'y.; Section of Obsteterics and Gynecology; J. A. Ireland, M. D., Louisville, Ky., chairman; S. H. Carlton, M. D., Seymour, Ind., Secretary; Section of State Medicine and Hygiene, J. W. Compton, M. D., Chairman, Evansville, Ind.; Section on Practical Medicine; J. H. Hibberd, M. D., Richmond, Ind., chairman.

J. W. COMPTON, M. D.,
Chairman of Committee of Arrangements.

THE FOLLOWING PAPERS are announced as the programme of the American Dermatological Association, for its First Annual Meeting, in Niagara, Sept. 4, 5 and 6, 1877:

1. On Molluscum contagiosum, by Dr. George Henry Fox, of New York. 2. Annual Address, by the President, Dr. James C. White, of Boston. 3. The Etiology of Cutaneous Diseases, by Dr. Lunsford P. Yandell, of Louisville, Ky. 4. On the Eczema marginatum of Hebra (tinea trichophytina cruris), as observed in America, by Dr. L. Duncan Bulkley, of New York. 5. The Pathology of Seborrhœa, by Dr. Arthur Van Harlingen, of Philadelphia. 6. Faulty innervation as a factor in Skin Disease, by Dr. Edward Wigglesworth, of Boston. 7. A case of true Prurigo (of Herba), by Dr. Robert Campbell, of New York. 8. On the Immunity of certain Mothers of children affected with Hereditary Syphilis, by Dr. James Nevins Hyde, of Chicago. 9. The Lymphatic Theory of Syphilitic infection, with a new view of the relation between the chancre and chancroid; and suggestions for the radical cure of Syphilis, by Dr. Wm. A. Hardaway, of St. Louis, Mo. 10. On the Relation of Impetigo Herpetiformis to Pemphigus, by Dr. C. Heitzmann, of New York. 11. On the Xeroderma of Hebra, by Dr. R. W. Taylor, of New York. 12. Case of an unusual form of Fragilitas Crinium, by Dr. Louis A. Duhring, of Philadelphia. 13. Two Cases of very late Hereditary Syphilis, by Dr. L. Duncan Bulkley, of New York. 14. The Pathological Histology of Psoriasis, by Dr. A. R. Robinson, of New York. 15. Auto-inoculation of Vegetable Parasites, and their non-identity, by Dr. Edward Wigglesworth, of Boston. 16. Affections of the testicle in Hereditary Syphilis, by Dr. R. W. Taylor, of New York. 17. Acute Conditions of disease excited by Iodide of Potassium, by Dr. Almon Brooks, of Chicago.

---

The Eighth Annual Meeting of The American Association for the Cure of Inebriety, will be held in the Washingtonian Home, Chicago, Ill., Sept. 12, 1877.

The following papers are promised:

"Manifestations of premature mental decay and nervous exhaustion, induced by inebriety and its treatment." By Dr. E. C. Mann, New York city.

"Morbid Appetites." By Dr. George Burr, Binghamton, N. Y.

"On the principles which should govern the management of inebriates, and the institutions needed to aid in their restoration." By Dr. N. S. Davis, Chicago, Ill.

"Nature and treatment of inebriety." By Dr. George M. Beard, New York City.

"Insanity and inebriety contrasted." By Dr. Joseph Parrish, Burlington, N. J.

"The obligations of the State respecting inebriety." By Dr. T. H. Evarts, Rushford, Minn.

"The curability of inebriety." By Dr. Albert Day, Boston, Mass.

"Persistent alcoholism." By Dr. C. W. Earle, Chicago, Ill.

"Inebriety and its symptomatology." By Dr. T. D. Crothers, Hartford, Conn.

"The work of managing inebriates." By Professor D. Wilkins, Chicago, Ill.

"The responsibility of the medical profession in the production of opium inebriety." By Dr. J. B. Mattison, Brooklyn, N. Y.

"Hereditary inebriety." By Dr. B. N. Comings, New Britain, Conn.

"The gradual reduction of opium in the treatment of opium inebriety." By Dr. C. T. Widney, St. Louis, Mo.

"Appetency for alcohol." By Dr. D. H. Kitchen, Binghamton, N. Y.

Other papers are promised, but the titles are not yet received.

The President's address will be on the disease of inebriety; one evening will be devoted to a public reception, in which addresses from prominent men are expected.

This promises to be one of the most important meetings ever held by the Association.

### AMERICAN PUBLIC HEALTH ASSOCIATION, 1877.

### Fifth Annual Meeting, Chicago, Sept. 25—27.

To The Editor of The Chicago Medical Journal and Examiner :—

The American Public Health Association will convene in Chicago. Ill., September 25th, for its Fifth Annual Meeting. It will, as usual, continue in session three days. The leading topics for discussion will be those which are becoming most practically important in the growing cities and towns of the great West, and the more dense populations of cities in the older States. The President of this Association, in his capacity as President of the State Board of Health of Illinois, fitly represents the interests of the State Sanitary Service as now provided in eight of the States west of the Alleghany Mountains. To meet the active members of those State Boards, and to take conference with them, and all others who join with this Association, in the studies and efforts for promoting the public health, and enhancing the value and certainties of human life, will be a privilege greatly appreciated by all who attend this meeting.

The city of Chicago presents, at one view, a most comprehensive field illustrating the problems of sanitary engineering, and the public health service in great cities; and its sanitary engineers are successfully working out the most important of these problems, while the medical profession and health officers are active in the studies and duties of preventive medicine. The city of the prairies is becoming the city of parks, and, in common with the thousands of treeless towns in the West, the people are surrounding themselves with trees as Nature's friends of health. The subject of Trees and Forests is among those to be reported upon and dis cussed. Sanitary Drainage and the Remedies for Natural Disadvantages of the Sites and Sanitary Topography of Cities and Villages will be one of the topics discussed at the opening of the session ; and important papers will be presented and discussed, relating to Preventive Measures against Scarlatina, Diphtheria, Trichina, and other destructive maladies; while other more general topics will be brought to the attention of the Meeting, it is expected, about in the order shown in the annexed list of titles. The names of the contributors to the proceedings will be announced at the proper time by the usual circular, and it is especially desired that the papers on the Geography of *Phthisis*, by Prof. Hosmer A. Johnson; on Sanitary and Medical Care of the Poor, by Prof. Edmund Andrews; that on the "Stamping Out" Process in Zymotic Pests, by Prof. H. M. Lyman, and that on Forests and Trees, by Dr. Andrews, may be followed by very thorough elucidation, and practical discussion. On behalf of the Executive Committee.

Elisha Harris, *Secretary*.

The Executive Committee has reccommended that, for this meeting, the leading discourses, papers and discussions shall be arranged under the following Division of Subjects :

I. Sanitary Topography and the Systematic Drainage and Sewerage of Cities and Villages.—Other Problems of Public Health.

II. Sanitary Control and Extermination of the Contagia of Typhoid (Enteric) Fever, Scarlatina, Small-Pox, etc.—Other Problems of Domestic Health.

III. Expert Testimony and the Pursuit of Exact Scientific Researches in Public Health Service.

Under these three general divisions, contributions are already promised upon the following topics, and, upon several of them, there will be discussions and written statements from original observation and experience :—

Sanitary Topography of Chicago, and the Artificial Remedies of Natural Disadvantages.

Proposed Sanitary Survey of the United States.

Problems old and new in the Sanitary Drainage and Sewerage of Chicago and other Western Cities.

Sanitary Geography of Phthisis Pulmonalis and other Pulmonary Diseases.

Sanitary value of Forests.

Garden City.

Problems of Sanitary Inspection. Safe-keeping and Transportation of Food Supplies—Animal and Vegetable.

Sanitary Lessons of the Great Fire in Chicago.

"Stamping Out" Scarlatina and the Extinguishment of Zymotic Diseases.

Sanitary Observations and Preventive Measures in regard to Diphtheria.

Teachings of 22 years' Records of Mortality from Croup, Diphtheria and Scarlatina in a City.

Miasmata of Dwellings and Dwelling-grounds.

Family Health and the Relations of Heredity, Habit and Hygiene to it.

Practicable Method for securing Complete and Authentic Records of the Causes of Death throughout the United States.

Sources and Propagation of Trichina, considered with reference to Sanitary Measures.

Hygienic Care of Sailors and other Water-men, upon the Coastwise and Inland Waters.

Sanitary Safety in Railway Traveling.

Expert Testimony.

Hygiene in the Prevention of Insanity.

Sanitary and Economical Importance of the Best Surgical and Medical Treatment of the Needy Poor.

Tests and Supervision of Milk-Supplies of Large Cities.

Health in Education, as viewed in each Grade of the Schools.

Points of Enumeration and Inquiry which the Interests of the Public Health require in the TENTH NATIONAL CENSUS.

Methods of Promulgating Sanitary Knowledge and Authorized Information in regard to Vital Statistics and Prevalent Diseases.

Judicial and other Aids of Law in Public Health Service.

Discourse on the Present State of Exact Knowledge of the Causation and Prevention of Destructive Diseases which most harm Mankind.

### Officers for the Year 1876–7.

President, John Rauch, M. D., Chicago, Ill; First Vice-President, Lewis H. Steiner, M. D., Frederick, Md; Second Vice President, E. M. Hunt, M. D., Metuchen, N. J; Secretary, Elisha Harris, M. D., New York, N. Y; Treasurer, J. Foster Jenkins, M. D., Yonker, N. Y.

#### ELECTED MEMBERS OF EXECUTIVE COMMITTEE.

John S. Billings, M. D., U. S. A; John M. Woodworth, M. D., D. C; J. L. Gibon, U. S. N; Hon. Jackson S. Schultz, N. Y; Dr. C. H. Olson, Mass; Dr. H. A. Johnson, Ill.

